# Ethnic-specific association of amylase gene copy number with adiposity traits in a large Middle Eastern biobank

**DOI:** 10.1038/s41525-021-00170-3

**Published:** 2021-02-09

**Authors:** Niccolo’ Rossi, Elbay Aliyev, Alessia Visconti, Ammira S. A. Akil, Najeeb Syed, Waleed Aamer, Sujitha S. Padmajeya, Mario Falchi, Khalid A. Fakhro

**Affiliations:** 1 Department of Twin Research and Genetics Epidemiology, King’s College London, London, UK; 2 Department of Human Genetics, Sidra Medicine, Doha, Qatar; 3 Department of Genetic Medicine, Weill-Cornell Medical College, Doha, Qatar; 4 College of Health and Life Science, Hamad Bin Khalifa University, Doha, Qatar

**Keywords:** Genetic association study, Clinical genetics

## Abstract

Studies assessing the impact of amylase genes copy number (CN) on adiposity report conflicting findings in different global populations, likely reflecting the impact of ancestral and ethnic-specific environment and lifestyle on selection at the amylase loci. Here, we leverage population size and detailed adiposity measures from a large population biobank to resolve confounding effects and determine the relationship between salivary (*AMY1*) and pancreatic (*AMY2A*) amylase genes CN and adiposity in 2935 Qatari individuals who underwent whole-genome sequencing (WGS) as part of the Qatar Genome Programme. We observe a negative association between *AMY1* CNs and trunk fat percentage in the Qatari population (*P* = 7.50 × 10^−3^) and show that Qataris of Arab descent have significantly lower CN at *AMY1* (*P* = 1.32 × 10^−10^) as well as less favorable adiposity and metabolic profiles (*P* < 1.34 × 10^−8^) than Qataris with Persian ancestry. Indeed, lower *AMY1* CN was associated with increased total and trunk fat percentages in Arabs (*P* < 4.60 × 10^−3^) but not in Persians. Notably, overweight and obese Persians reported a significant trend towards dietary restraint following weight gain compared to Arabs (*P* = 4.29 × 10^−5^), with *AMY1* CN showing negative association with dietary self-restraint (*P* = 3.22 × 10^−3^). This study reports an association between amylase gene CN and adiposity traits in a large Middle Eastern population. Importantly, we leverage rich biobank data to demonstrate that the strength of this association varies with ethnicity, and may be influenced by population-specific behaviors that also contribute to adiposity traits.

## Introduction

Salivary (AMY1) and pancreatic (AMY2A) amylase enzymes are responsible for starch digestion, which begins in the oral cavity and continues in the small intestine. The *AMY1 and AMY2A* genes show extensive copy number (CN) variability in humans, with a reported number of gene copies ranging from 2 to 18 for *AMY1*, and from 0 to 8 for *AMY2A*^1–4^. It has been shown that CN distribution at the salivary amylase gene is significantly variable between populations, with the number of copies of *AMY1* reflecting a biological adaptation to traditionally high-starch or low-starch diets throughout evolution^5^.

In 2014, we first reported an association between reduced *AMY1* CN and increased body mass index (BMI) and obesity risk using 6200 individuals of European and Asian ancestries^6^, although subsequent studies attempting to replicate this association yielded conflicting results. For instance, association with *AMY1* CN was not replicated in 4000 individuals of European ancestry, including people selected for being at the extremes of the BMI distribution^3^, as well as in a case-control study of 932 Chinese and 145 Malay samples^7^, and in 1400 participants from the UK 1958 Birth Cohort^8^. On the other hand, studies specifically analyzing obesity in children and young adults supported the association of BMI with amylase gene copy number in French^9^, Mexican^10^, and Italian children^11^, as well as in females with early-onset obesity from Finland^12^.

These studies highlight the complexity of studying an endpoint which has both genetic and environmental components, and suggest that differences in ethnicity, environment and food preferences may further influence the manifestation of this complex phenotype in the setting of genetic susceptibility. Indeed, previous works exploring the relationship between *AMY1* CN and diet revealed a significant effect of the interaction between *AMY1* CN and starch intake on BMI in 4800 nondiabetic adults from Sweden^13^, and greater weight and central adiposity loss following randomized low-calorie diet interventions among carriers of the allele rs11185098-A (a proxy of higher *AMY1* CN and activity^3^), compared to noncarriers, among 692 Europeans from The POUNDS Lost Trial^14^. Taken together, these studies suggest that environmental factors, and particularly dietary choices, may play a role in modulating the observed association between *AMY1* CN and adiposity.

Here, we report a large-scale association study between CN at the amylase genes (*AMY1* and *AMY2A*) and adiposity traits in a large Middle Eastern cohort. Specifically, we combined CN inference from high-coverage (30×) whole-genome sequencing (WGS) with phenotypic traits related to adiposity traits and behaviors, collected for almost 3000 subjects as part of the Qatar Biobank^15^. Our findings help explain trans-ethnic differences in the effect of amylase CN on adiposity and introduce a role for subpopulation-specific traditional dietary and lifestyle choices in determining the strength of association between amylase and adiposity in global populations.

## Results

### Distribution of adiposity traits in the Qatari population

The Qatar Biobank (QBB) collected a wide range of traits, biochemical measurements, and lifestyle questionnaires from adult Qatari nationals and long-term residents^15^. While many of these traits will be available for future studies, we focused this first analysis of QBB data on adiposity traits, specifically: BMI, total fat percentage, and trunk fat percentage.

**Fig. 1:**
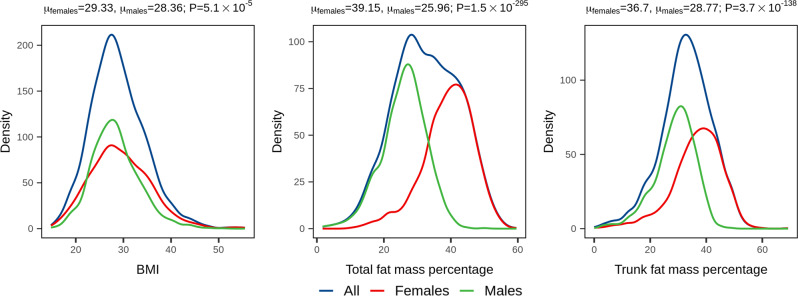
Distribution of adiposity traits. Density plots showing BMI, total and trunk fat percentages distribution in the Qatari population (*N* = 1471 and 1464 females and males, respectively). The mean trait value (µ), stratified by sex, was evaluated by means of the Wilcoxon’s test, and p-values are reported above each panel.

Among the 2935 individuals for whom genomic data was available as part of the Qatar Genome Programme (QGP) pilot phase, 1464 were females and 1471 males. Measures of BMI, total and trunk fat percentages were normally distributed with means (SD) of 28.9 (5.7), 32.7 (9.7) and 32.8 (9.1), respectively (Table 1, Fig. 1). Mean age was not statistically different between sex groups (Wilcoxon’s *P* > 0.05). Conversely, all the adiposity traits under study were significantly higher in female than in male subjects (Table 1, Fig. 1; Wilcoxon’s *P* < 5.08 × 10^−5^), with the strongest difference being observed for total fat percentage (Wilcoxon’s *P* = 1.54 × 10^−295^).

**Table 1:** Adiposity traits of 2935 QBB individuals sequenced as part of the QGP pilot phase.

	All	Females	Males	*P*
*N*	2935	1471	1464	—
Age (years)	39.11 ± 12.03	39.50 ± 12.62	38.72 ± 11.42	>0.05
	[29.00–48.00]	[29.00–49.00]	[30.00–46.00]	
BMI (kg/m^2^)	28.86 ± 5.71	29.33 ± 6.28	28.36 ± 5.00	5.08 × 10^−5^
	[24.90–32.40]	[24.90–33.40]	[24.90–31.30]	
Total fat (%)	32.69 ± 9.72	39.15 ± 7.56	25.96 ± 6.68	1.54 × 10^−295^
	[25.70–40.20]	[34.8–44.5]	[22.00–30.33]	
Trunk fat (%)	32.82 ± 9.07	36.70 ± 8.93	28.77 ± 7.27	3.71 × 10^−138^
	[27.50–38.90]	[31.90–43.00]	[24.67–33.70]	

Reported here are means, standard deviations, 1st–3rd interquartile ranges (in brackets), and statistical differences between sex groups (Wilcoxon’s test *p*-values are reported).

### Range of *AMY1* and *AMY2A* copy number variability in the Qatari population

We first aimed to estimate the range of discrete CN variation at human salivary (*AMY1*) and pancreatic (*AMY2A*) amylase genes in the Qatari population by coverage analysis of WGS data from 2935 individuals from the QBB (see “Methods” section).

Following post-mapping quality control of reads from whole-genome sequencing data (see “Methods” section), we found that the percentage of correctly aligned reads ranged from 90 to 97%, for *AMY1* (mean = 95.2%, median = 95.3%, interquartile range [IQR] = 94.7–95.8%), and from 90 to 99%, for *AMY2A* (mean = 94.5%, median = 94.7%, IQR = 94.2–95.0%; Supplementary Fig. 1). In addition, the average per-individual percentage of reads with mapping quality score >20 and uniquely mapping to both *AMY1* and *AMY2A* was 99.99%, corresponding to a probability of inaccurate mapping of at most 0.01 (Supplementary Fig. 1).

**Fig. 2:**
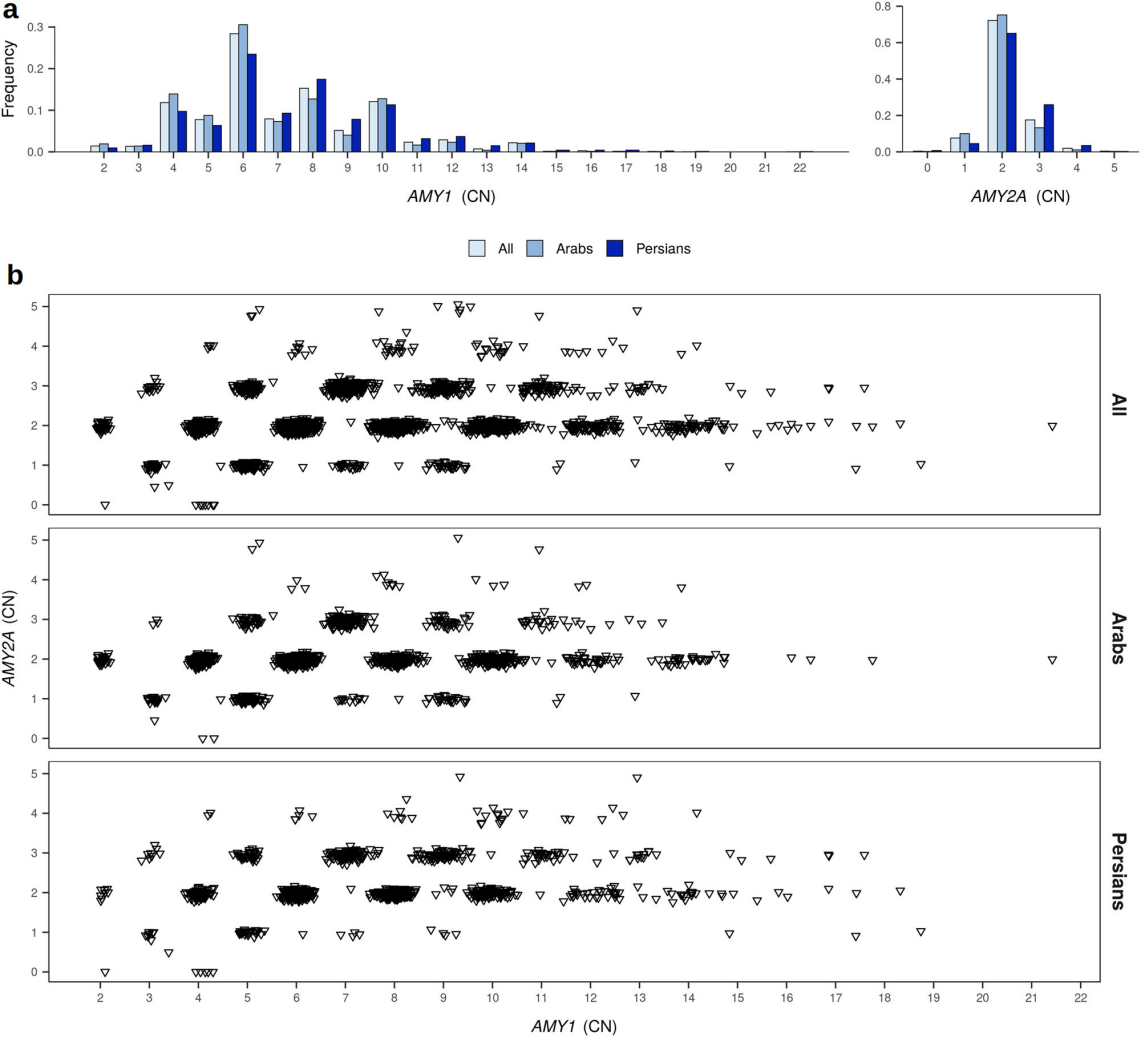
*AMY1* and *AMY2A* variation in the Qatari population. **a** Histograms showing the distribution of estimated copy numbers of *AMY1* and *AMY2A*, obtained from coverage analysis of whole-genome sequencing data in all the Qatari population (*N* = 2935, white), and Arabs (*N* = 1518, light blue) and Persians (*N* = 948, dark blue) from Qatar. **b** In the Qatari population, *AMY2A* and *AMY1* show parity: when *AMY2A* copy number is odd, so is *AMY1* copy number.

In the Qatari population, *AMY1* gene copy number ranges from 2 to 22, *AMY2A* from 0 to 5, and *AMY2B* from 1 to 5 copies. Consistent with previous reports^1,3,6,16^, the number of copies of *AMY1* and *AMY2A* were significantly correlated to each other (polychoric correlation estimate ρ = 0.28, *P* < 2.22 × 10^−308^; Fig. 2b), with 72% of the Qatari population carrying an even diploid number of *AMY1* genes and two copies of *AMY2A*.

The number of copies of *AMY2A* and *AMY2B* showed high correlation (Pearson’s ρ = 0.81, *P* < 2.22 × 10^−308^), corresponding to 90% of QBB samples carrying the same number of copies of these two genes. In 88% of the discordant CN calls at *AMY2A* and *AMY2B*, *AMY2A* arbored CN variations, whereas *AMY2B* was copy-invariable (CN = 2). This is consistent with previous studies reporting 95% of haploid similarity between *AMY2A* and *AMY2B* CN^2,3^, and substantial higher CN variation (24% vs. 10%, respectively) at *AMY2A* compared to *AMY2B*^2^. Due to the extensive overlap between *AMY2A* and *AMY2B* CN, and the higher variability observed at the *AMY2A* locus compared to *AMY2B*, when these were CN-discordant, *AMY2B* was excluded from subsequent analyses.

We further investigated the presence of damaging loss-of-function (LoF) variants within the coding region of *AMY1* and *AMY2A* and which may affect the number of active copies of the genes and therefore skew the results (see “Methods” section); however we found no variants with putative disruptive effects (i.e., stop gained, frameshift, splice variants or in-frame deletions). Indeed, the frequency of LoF variants in *AMY1* and *AMY2A* is reported to be rare (MAF < 1%) in the general population based on the Genome Aggregation Database (gnomAD)^17^.

### Validation of amylase copy number estimates

We estimated *AMY1* and *AMY2A* CN on 40 samples using digital droplet PCR (ddPCR; Methods), identifying only six discrepancies with respect to the CNs estimated with the Carpenter et al.’s approach^1^ used here (five for *AMY1* calls and one for *AMY2A* calls, Supplementary Table 1), therefore confirming a very high accuracy (74/80 = 92.5%) of our calls across both loci. Notably, four out of five instances of discrepancy at *AMY1* deviated from the ddPCR results for a single copy, with consequent deviation from shared odd/even parity between *AMY1* and *AMY2A* CN. Such a deviation is reported to have a low frequency in the general population^3^, and was avoided in our analysis, where conditional rounding was applied.

We used AMYCNE^18^ to further validate the *AMY1* CN inferred using the approach described by Carpenter et al.^1^ and observed a strong agreement between the two approaches (Pearson’s ρ = 0.98; *P* < 2.22 × 10^−308^; *N* = 2935, Supplementary Fig. 2).

### Association of amylase genes CNs with adiposity in the Qatari population

Anthropometric measurements of adiposity (BMI, total fat percentage, and trunk fat percentage) were tested for association with *AMY1* and *AMY2A* CN in 2935 individuals from Qatar using PopPAnTe^19^, including sex, age at the sample collection, and the first 10 PCs from genome-wide data as covariates (see “Methods” section). At a Bonferroni-adjusted significance threshold of 8.33 × 10^−3^ (see “Methods” section), *AMY1* appeared significantly associated with trunk fat percentage (β = −0.02, SE = 0.01; *P* = 7.50 × 10^−3^; Table 2). Conversely, no significant association was observed for *AMY2A* (*P* > 0.05; Table 2).

**Table 2:** Associations between amylase copy number and adiposity traits in the Qatari population.

Gene	Trait	*N*	β	SE	*P*
*AMY1*	BMI (kg/m^2^)	2908	−0.02	0.01	3.26 × 10^−2^
*AMY1*	Total fat (%)	2740	−0.01	0.01	1.10 × 10^−2^
*AMY1*	Trunk fat (%)	2726	−0.02	0.01	7.50 × 10^−3^
*AMY2A*	BMI (kg/m^2^)	2897	−0.07	0.03	2.92 × 10^−2^
*AMY2A*	Total fat (%)	2729	−0.04	0.02	0.108
*AMY2A*	Trunk fat (%)	2715	−0.06	0.03	0.050

Association testing between amylase genes CN and inverse normalized adiposity traits was performed in 2935 Qataris using PopPAnTe, which takes into account individuals’ kinship, age, sex and the first 10 PCs from genome-wide data as covariates. The table reports the number of samples included in the tested datasets (N), the effect sizes (β) with their standard errors (SE), and the association *p*-values (*P*).

### Qatari genetic subpopulations

**Fig. 3:**
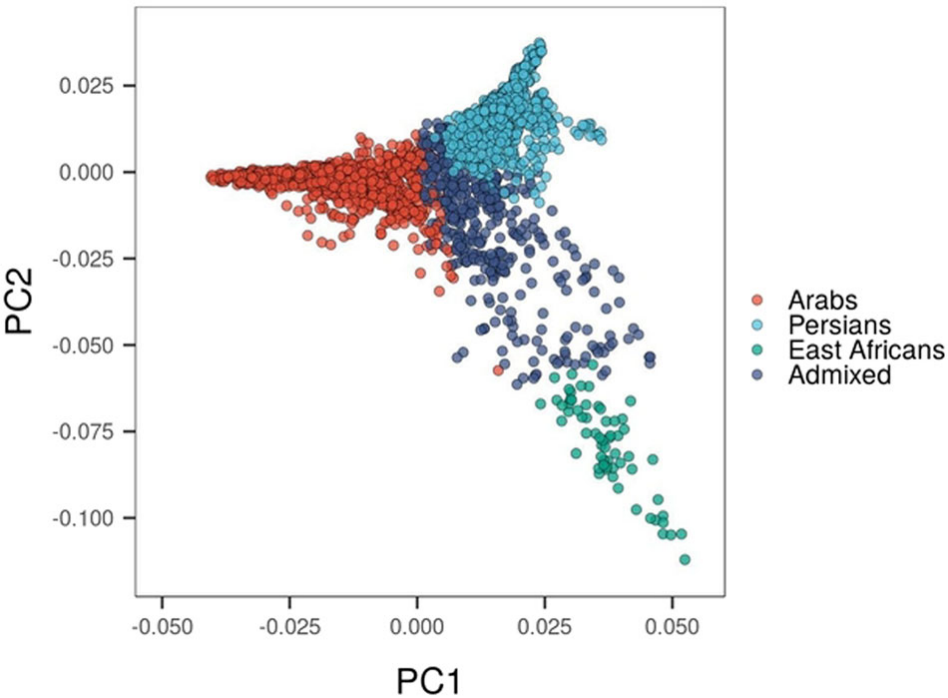
Principal component analysis. Scatterplot of the first two principal components assessed on ancestry-informative SNPs of 2,935 individuals from Qatar (see “Methods” section). Individual data points are color-coded according to previously described major ethnic ancestry: Arabs (red), Persians (light blue), and East Africans (green). Admixed individuals (purple) are those whose majority ancestry cannot be assigned to one group.

Since the Qatari population includes subjects of different ancestries, we further sought to distinguish them within our sample and assess their contribution to the observed association between *AMY1* and adiposity. To identify the underlying genetic ancestries, we performed principal component analysis (PCA) using ancestry informative markers^20,21^ and identified individuals clustering with three major Qatari subpopulations (namely, Bedouin Arabs, Persians and East Africans; see “Methods” section, Fig. 3). We excluded from further analyses East Africans, because of their small sample size (*N* = 63), along with admixed individuals (*N* = 406) which could not be assigned to a majority ethnic ancestry. Persians (*N* = 948) and Arabs (*N* = 1518) were thus selected for subsequent study.

**Fig. 4:**
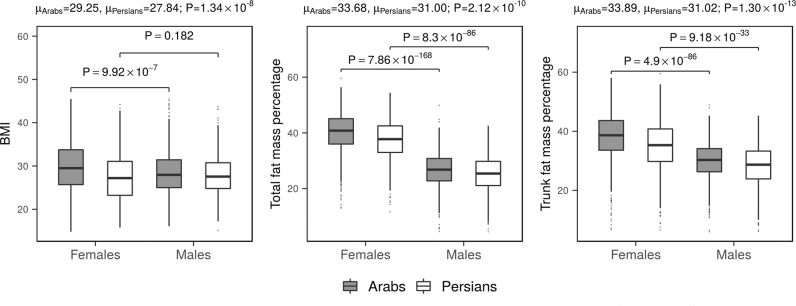
Distribution of adiposity traits in Arabs and Persians from Qatar. Boxplots showing BMI, total and trunk fat percentages distribution in Arabs (*N* = 1518, gray) and Persians (*N* = 948, blue). The center line of the boxplots indicates the median, limits of the box indicate the 25th and 75th percentile. The whiskers represent either 1.5 times the interquartile range (IQR) or the maximum/minimum data point if they are within 1.5 times the IQR. The mean trait value (µ) stratified by ethnic group and the p-values, evaluated by means of the Wilcoxon’s test, are reported above each panel. Sex-stratified *p*-value within each ethnic group are reported within each panel.

To avoid further demographic confounders, we investigated but did not find differences between the two groups in age (µ_Persians_ = 40,3; µ_Arabs_ = 39.1; Wilcoxon’s *P* = 0.02) or sex distribution (*χ*^2^ *P* > 0.05). Despite the similar make-up of the two groups, Qataris with Persian ancestry appeared to have an overall healthier adiposity profile than those with Arab ancestry, as measured by BMI (µ_Arabs_ = 29.25; µ_Persians_ = 27.84; Wilcoxon’s *P* = 1.34 × 10^−8^), total fat (µ_Arabs_ = 33.68; µ_Persians_ = 31.00; *P* = 2.1 × 10^−10^) and trunk fat percentages (µ_Arabs_ = 33.89; µ_Persians_ = 31.02; *P* = 1.3 × 10^−13^), all of which were significantly lower in Persians as compared to Arabs (Fig. 4). Furthermore, consistent with the distribution from the overall group, within each ethnic group total and trunk fat percentages were remarkably higher in females than males, though this difference was less pronounced in Persians (Wilcoxon’s *P*_Arabs_ < 4.90 × 10^−86^; *P*_Persians_ < 9.18 × 10^−33^; Fig. 4). Conversely, females were observed to have significantly higher BMI than males (*P* = 9.90 × 10^−7^; Fig. 4) only among Arabs.

### *AMY1* and *AMY2A* CNs are higher in Qataris with Persian ancestry

*AMY1* and *AMY2A* CNs showed significant correlations in both Arabs and Persians (polychoric correlation estimate ρ_Arabs_ = 0.27, *P* = 4.57 × 10^−315^; ρ_Persians_ = 0.29, *P* = 1.91 × 10^−201^; Fig. 2b), with 75 and 65% of the Arab and Persian subpopulation, respectively, carrying an even number of *AMY1* genes and two copies of *AMY2A*.

We observed significant differences in CN distribution at *AMY1* and *AMY2A* between Persians and Arabs (*χ*^2^ test *P* = 7.23 × 10^−12^ and *P* = 9.15 × 10^−21^, respectively), with Persian subjects appearing to have alleles with a higher number of copies of *AMY1* (µ_Arabs_ = 6.92 copies; µ_Persians_ = 7.60 copies; Wilcoxon’s test *P* = 1.32 × 10^−10^; Fig. 2a), as well as *AMY2A* (µ_Arabs_ = 2.06; µ_Persians_ = 2.27; Wilcoxon’s test *P* = 7.49 × 10^−21^; Fig. 2a).

Differences in *AMY1* and *AMY2A* CNs distribution between Arabs and Persian were supported by differences in estimated haplotypes frequencies at both loci (Supplementary Methods; Supplementary Results, Supplementary Fig. 3).

### Genetic ancestry at the *AMY1* locus

To rule out the possibility that the amylase locus may not reflect the overall genetic ancestry assigned to each individual based on genome-wide markers, we performed a further PCA using SNPs and indels located within a 5-MB window surrounding *AMY1* (see “Methods” section). Our analysis confirmed that individuals of Arab and Persian ancestry were still well separated in this novel principal component space (Supplementary Fig. 4), while also highlighting the presence of a small subset of outliers (*N*_Arabs_ = 75, *N*_Persians_ = 34), likely reflecting finer ancestry substructures within these main ancestry groups. Notably, we investigated whether the exclusion of these individuals would influence the association signals identified in our primary analysis, and found negligible differences (see “Methods” section, Supplementary Table 2).

### Association between *AMY1* CN and adiposity traits in Arabs and Persians

Total fat percentage, BMI, and trunk fat percentage were tested for association with *AMY1* CN in 948 Persians and 1518 Arabs from Qatar using PopPAnTe^19^. Sex and age at sample collection were included as covariates, as well as the first 10 PCs from genome-wide data (see “Methods” section).

**Fig. 5:**
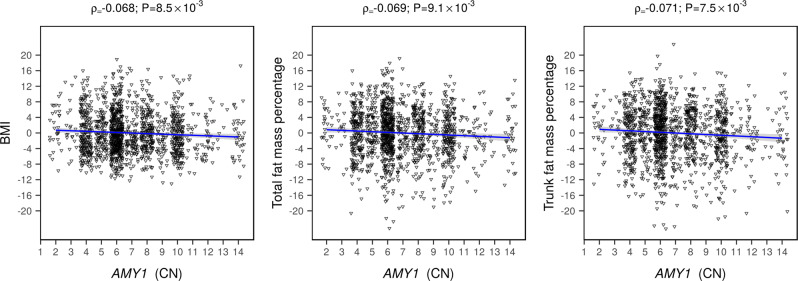
*AMY1* CN association with adiposity in Arabs from Qatar. The scatter plots depict the correlation between salivary amylase copy number (*x*-axis) and sex-corrected and age-corrected measures of adiposity (*y*-axis). Pearson’s correlation coefficients (ρ) and *p*-values are reported above each panel.

At a Bonferroni-adjusted significance threshold of 8.33 × 10^−3^ (see “Methods” section), we observed negative associations between *AMY1* CNs and both total and trunk fat percentages in Arabs (*P* < 4.60 × 10^−3^; Table 3, Fig. 5). In addition, lower *AMY1* CNs was nominally associated with increased BMI in Arabs (*P* = 8.40 × 10^−3^). Conversely, no significant association (*P* > 0.05) was observed in Persians; however, the direction of association of *AMY1* with adiposity was concordant between ethnic groups (Table 3).

**Table 3:** Associations between *AMY1* copy number and adiposity traits in Persians and Arabs from Qatar.

Ancestry	Trait	*N*	β	SE	*P*
Arabs	BMI (kg/m^2^)	1505	−0.03	0.01	4.60 × 10^−3^
Arabs	Total fat (%)	1432	−0.02	0.01	8.40 × 10^−3^
Arabs	Trunk fat (%)	1425	−0.03	0.01	4.40 × 10^−3^
Persians	BMI (kg/m^2^)	944	0.00	0.01	0.804
Persians	Total fat (%)	879	−0.01	0.01	0.300
Persians	Trunk fat (%)	874	−0.01	0.01	0.320

Association testing between *AMY1* CN and inverse normalized adiposity traits was performed in 1518 Arabs and 948 Persians from Qatar using PopPAnTe, which takes into account individuals’ kinship, age, sex and the first 10 PCs from genome-wide data as covariates. The table reports the number of samples included in the tested datasets (N), the effect sizes (β) with their standard errors (SE), and the association *p*-values (*P*).

In addition, case-control analysis of 593 obese (BMI ≥ 30 kg/m^2^) and 357 normal weight (BMI < 25 kg/m^2^) Arab individuals from the same dataset highlighted that higher *AMY1* CN was indeed inversely associated with obesity risk (OR = 0.93, 95% CI = [0.88–0.98]; *P* = 0.01).

### Contribution of population specific behaviors to *AMY1* association with adiposity

Both *AMY1* copy number variability and its contribution to adiposity are thought to arise as a product of the adaptive interaction between *AMY1* and environmental exposure to starch throughout evolution^5^. As a consequence, it is prudent to consider that diverse ancestral environments and differences in lifestyle choices (e.g., dietary behavior) may have impacted *AMY1* CNVs association with adiposity in Persian and Arab Qataris.

Among study subjects, 1805 individuals (1148 Arabs and 657 Persians) were overweight or obese (BMI ≥ 25 kg/m^2^). Notably, in reviewing the QBB intake questionnaires, we found that 1013 of these declared to have modified their diet during the year of data collection because of excess weight and its related comorbidities (e.g., high cholesterol, hypertension, and diabetes). Furthermore, we observed by multiple logistic regression (see “Methods” section) a significant difference between Arabs and Persians in the tendency towards dietary self-restraint following weight gain, with 54 and 60% of overweight or obese Arabs and Persians restraining their diet, respectively (*P* = 4.29 × 10^−5^). In the same analysis, sex also appeared to be a significant (*P* = 1.36 × 10^−6^) determinant of dietary behaviors in overweight or obese subjects from Qatar, with 60% of overweight females (58 and 65% in Arabs and Persians, respectively) displaying dietary restraint, as compared to only 52% of overweight males (49 and 56% in Arabs and Persians, respectively). Interestingly, higher *AMY1* copy number was negatively associated with diet restraint in overweight subjects (OR per estimated unit copy increase = 0.94, 95% CI = 0.91–0.98; *P* = 3.22 × 10^−3^).

### Effect of ethnicity on the association between *AMY1* and adiposity traits and on dietary restraint

It could be hypothesized that if *AMY1* CN segregates with ancestry, such that increased Arab ancestry corresponds to decreased *AMY1* CN, and at the same time genetic factors other than *AMY1* increase the risk of adiposity in Arabs, such that increased Arab ancestry corresponds to increased adiposity, then these two independent events would generate a spurious association between *AMY1* CN and adiposity traits. To investigate this further, we explored the relationship between varying degrees of ancestry, as defined using the first principal component (PC1) values (see “Methods” section) and both *AMY1* CN and adiposity traits.

After accounting for age and sex, adiposity traits showed significant negative associations with PC1 among both individuals of Arab ancestry (β = −4.66, SE = 2.47, *P* = 0.06; β = −4.85, SE = 1.79, *P* = 6.88 × 10^−3^; β = −6.31, SE = 2.12, *P* = 3.02 × 10^−3^, for BMI, total and trunk fat and total fat percentages, respectively) and Persian ancestry (β = −18.53, SE = 6.18, *P* = 2.77 × 10^−3^; β = −14.27, SE = 4.75, *P* = 2.73 × 10^−3^; β = −18.90, SE = 5.79, *P* = 1.13 × 10^−3^, for BMI, total and trunk fat and total fat percentages, respectively; Supplementary Fig. 5). Conversely, *AMY1* CN did not associate with PC1 in Persians (*P* = 0.23), and only weakly in Arabs (β = −13.57, SE = 6.57, *P* = 0.04; Supplementary Fig. 5), where an increased Arab ancestry actually associated with higher copy number, *i.e*., in the opposite direction compared to our original association results. Therefore, our main findings do not appear to be biased by potential co-segregation of adiposity and *AMY1* CN with ancestry.

Interestingly, while no difference in the association between *AMY1* and adiposity was observed between subgroups of subjects characterized by varying degrees of Persian ancestry, the higher the Arab ancestry, the stronger the association with all adiposity traits (see “Methods” section, Supplementary Fig. 6). To explore what could drive this behavior, we evaluated the heritability of the three adiposity traits at varying degrees of ancestry (see “Methods” section). While, in general, the heritability of all adiposity traits was higher among Persians (42–44%) compared to Arabs (19–23%), Arabs (but not Persians) showed decreased heritability of these traits with increased ancestry (Supplementary Fig. 6). This was not driven by a decreased number or strength of pairwise kinship between subjects with strongest Arab ancestry, which might have deflated the heritability estimate. Instead we hypothesized that this could have been caused by ethnic-specific behavioral or environmental factors. We further assessed the association between PC1 and dietary behavior, finding a significant association between the degree of Arab ancestry and decreased tendency towards dietary restraint (*P* = 1.26 × 10^−5^; Supplementary Fig. 7), while dietary restraint behavior did not change between subgroups of subjects characterized by varying degrees of Persian ancestry (*P* > 0.05). Consistent with this finding, we observed a positive association between *AMY1* CN and dietary restraint with increased Arab ancestry adjusting for age, sex and BMI, compared to Persians subjects (Supplementary Fig. 8).

## Discussion

Elucidating the relationship between genetic architecture and obesity-related traits is of fundamental scientific and public-health concern, primarily due to the chronic comorbidities associated with obesity and the increased risk of premature mortality across various human populations^22^. The national STEPwise survey conducted in Qatar in 2012 reported an obesity epidemic (>70% of the adult population had a BMI ≥ 25 kg/m^2^), accompanied by an alarming rate of comorbidities in the affected population, such as hypertension (32.9% of respondents aged 18–64) and type II diabetes (17.6% of men and 15.9% of women)^23^.

While previous works have investigated the association of SNPs, epigenetic markers and obesity in the Gulf Arab world^24–27^, no studies to date have uncovered copy number variants influencing adiposity in this population.

Here, we discretely genotyped the range of copy number variation at *AMY1* and *AMY2A* genes through coverage analysis of 30× whole genome sequencing data in 2935 individuals from Qatar, and showed that (1) copy number at both genes has similar distribution and correlation to previous reports^1,3,6,16^, and (2) *AMY1* CN is negatively associated with trunk fat percentage.

Putative mechanisms by which *AMY1* may be linked to obesity involves its role in modulating starch intake, sweet-taste perception, and carbohydrate digestion^28,29^ and gut microbiome composition^30,31^. It has been reported that subjects expressing lower *AMY1* levels show higher blood glucose and delayed insulin response following starch ingestion^28^, suggesting that carriers of lower *AMY1* CN may be at higher risk of developing insulin resistance if chronically relaying on starch-rich diets. In addition, increased ketonuria is associated with low serum amylase^32^ which further supports the hypothesis that individuals with lower *AMY1* CN may not be fully adapted to a carbohydrate-based diet. Indeed, a combined mass-spec and NMR metabolomics study of homogeneous and age-matched normal weight French adult females highlighted increased reliance on lipid *vs* glucose metabolism for energy production in low CN carriers at *AMY1*^33^.

Studies exploring how the human gut microbiome responds to *AMY1* CN variation yielded conflicting results. For instance, it has been shown that gut microbiome of individuals carrying high *AMY1* copies is enriched in microbes involved in complex polysaccharide degradation belonging to the Prevotella genus^30^, and that high Prevotella/Bacteroides ratio favors weight loss in response to dietary interventions^34,35^. Conversely, Poole and colleagues reported adiposity gain in germ-free mice following fecal microbiota transplantation from carriers of high *AMY1* CN^31^.

Whereas the hypothesis that high *AMY1* CN promotes obesity by modulating starch metabolism has still to be fully proven, it is known that *AMY1* CN has followed different evolutionary trajectories between populations, as a function of the variability in starch content across traditional diets^5^. Given the ethnic heterogeneity of Qataris from QBB^20,21^, we sought to dissect possible ancestry-specific contributions to *AMY1* CN association with adiposity in Qatar. Interestingly, we found that Arabs from Qatar (and females in particular) have, on average, unhealthier adiposity profiles, and significantly lower *AMY1* and *AMY2A* CNs (*P*_*AMY1*_ = 1.32 × 10^−10^ and *P*_*AMY2A*_ = 7.49 × 10^−21^, respectively) compared to Qataris of Persian ancestry. Higher *AMY1* and *AMY2A* CN in Persians compared to Arabs from Qatar are consistent with differences in ancestral diets across the two ethnic groups. Indeed, whereas the traditional diet of populations with a history of nomadic pastoralism (e.g., Bedouin Arabs) relies extensively on proteinaceous resources and simple saccharides, starchy food resources traditionally comprise a substantial portion of the diet in traditional agricultural societies, such as Persians^5^. Higher ancestral *AMY1* and *AMY2A* CN in Persians from Qatar compared to Arab may have resulted in increased resilience, as supported by their more favorable adiposity profile, to dietary changes introduced by the rapid growth and occidentalization of Qatar brought about by newfound oil-wealth, which paralleled the increase of obesity prevalence^36^.

Despite showing similar negative trends, the association between *AMY1* CNs and measures of adiposity reached statistical significance only in Arabs, possibly due to lack of statistical power when testing Persians, given the smaller sample size or, indeed, population-specific effects.

We leveraged responses from the QBB on self-reported dietary restraint and observed a significant difference between Arabs and Persians in their tendency towards dietary restraint following weight gain (*P* = 4.29 × 10^−5^), with 60% of overweight or obese Persians restraining their diet compared to 54% of their Arab counterpart. In addition, we showed that increased Arab ancestry is associated with both decreased tendency towards dietary restraint following weight gain and increased association strength between *AMY1* CN and dietary behavior, thus providing further evidence of the complexity of the association between *AMY1* and adiposity, as it can be affected by population-specific environmental factors. This is supported by previous studies reporting a significant effect of the interaction between *AMY1* CN and dietary starch intake on BMI^37^, and an association between *AMY1* and BMI in overweight individuals on dietary intervention^14^. Unfortunately, food frequency questionnaires were not available to us for this cohort, and therefore we could not assess the contribution of dietary starch intake to the association between *AMY1* CN and obesity in the Qatari population. Additional data on dietary variables is needed in order to dissect the specific environmental contributions underlying these observations.

Taken together, these findings could partially explain the fluctuating success rate of studies attempting to replicate the associations between *AMY1* CN and obesity in adults, as compared to more consistent results observed in pediatric cohorts, where lifestyle choices and age-dependent gene-environment interactions should be less pronounced.

In conclusion, here we investigate the association between adiposity traits and discrete copy number variation from WGS at highly polymorphic loci in the Qatari population. We demonstrated that within this single national population of the Middle East, the strength of genetic associations may differ significantly across individuals with different ethnic ancestries. To help explain this disparity, we also demonstrate the power of a large population biobank such as QBB, rich with genomic and phenotypic information, not only to drive discoveries of novel associations in a part of the world that is relatively understudied in the literature, but to provide plausible explanations for such discrepancies.

## Methods

### Cohort description

The Qatar Biobank (QBB) is a national population-based medical health initiative started in 2012 with the aim of collecting samples and information on health and lifestyle from 60,000 Qatari adult nationals and long-term residents^15^. All subjects are enrolled in the Qatar Biobank via informed consent, and their genetic and phenotypic data made available for use in research studies. Anthropometrics details used in the current study, including BMI and body composition, were measured using the Seca stadiometer and Seca Bio Impedance Analysis (Seca GmbH and Co. KG, Hamburg, German) at Qatar Biobank facility at Hamad Medical City. Outliers (measurements greater than 3 standard deviations the dataset mean) were removed, and the data inverse-normal transformed.

The Qatar Biobank study was approved by the Qatar Biobank Institutional Review Board (IRB) Committee (IRB protocol E/2017/RES-ACC-0032/0002).

### Whole-genome sequencing data

Subjects from the QBB were sequenced as part of the QGP. Whole-genome sequencing was performed on 2935 blood samples using Illumina HiSeq platform (150-bp paired-end) to an average coverage of 30×. Sequence reads were aligned to the Human Reference genome version GRCh37 using bwakit (v. 0.7.11).

### Amylase gene copy number estimation

*AMY1* and *AMY2* CNs were estimated through sequencing coverage analysis by comparing the number of reads mapping to *AMY1* and *AMY2* genes to a reference local GC-matched and copy invariant region, as described in ref. ^1^. Read counts were extracted from WGS bam files using samtools^38^ (v. 1.6) with the command *samtools view –c*, defining the regions intervals with a corresponding *bed* file (coordinates based on GRCh37 genome assembly).

*AMY1* corresponding region spans over 20 kb and includes the *AMY1* gene plus two surrounding near-identical DNA sequences (chr1:104190000–104210000, 104227213–104247214 and 104284138–104304150, respectively). Local 20 kb reference region intervals were: chr1:104059996–104070000 and chr1:104460001–104469995. No quality threshold was applied for filtering reads mapping to this region, being the repetitive region responsible for low local mapping quality scores, as suggested in^1^.

*AMY2A* CN was estimated counting reads mapping to the interval chr1:104153700–104161939, which includes exons 1–3 of *AMY2A*, and to its local copy-invariant comparator interval (chr1:104045000–104085000). A minimum mapping quality threshold of 20 was applied (*samtools* option: -q 20).

*AMY2B* CN was estimated counting reads mapping to the interval chr1:104114335–104135000 and to its local copy-invariant comparator interval (chr1:104000000–104100000 and chr1:104304000–104500000). A minimum mapping quality threshold of 20 was applied (samtools option: -q 20).

Local read density was calculated for *AMY1, AMY2A, AMY2B*, and their reference regions, dividing the absolute read count by the region size (*e.g*., the total number of reads mapping to *AMY1* region divided by 40k). Read densities were then normalized by their reference region read densities to obtain an estimate of their copy number state. Finally, raw CN estimates were assigned to integers by conditional rounding. More in detail, *AMY2A and AMY2B* read densities were first rounded to the nearest integer. *AMY1* read densities were then rounded to the nearest even number if *AMY2A* CN was even, and to the nearest odd number if *AMY2A* CN was odd.

Polychoric correlation between CNs was estimated using *polycor* R package (v. 0.7–10; *polychor* function).

### Evaluation of reads alignability at the amylase locus

We assessed the rate of misalignment of sequences mapping to the *AMY1* and *AMY2* genes by evaluating the fraction of properly paired reads (i.e., read pairs mapping with correct orientation with respect to one another, and with expected insert size) mapping to either *AMY1* or *AMY2A* having unique alignment. Alignability was estimated on the same genomic regions used for CN calling at *AMY1* and *AMY2A*. Within individual BAM files, uniquely mapping reads were defined based on the absence of the ‘XA:Z:’ and ‘SA:Z:’ tags (flagging secondary alignments and split reads, respectively) and the “*” score (flagging unmapped reads) in the mapping quality (MAPQ) field generated by bwa-mem (bwakit v. 0.7.11). Further, we evaluated the distribution of the mapping quality of reads aligned to the *AMY1* and *AMY2A* genes.

### Validation of copy number estimates

We used Droplet Digital^TM^ PCR^39^ (ddPCR, Bio-Rad) to experimentally validate CN estimates at the *AMY1* and *AMY2* loci, following a previously published protocol^3^. PCR primers and fluorescent probes used in this study are reported in Supplementary Table 3. All probes targeting the *AMY1* (two independent probes) and *AMY2A* (one probe) genes were labeled with FAM, whereas control probes with HEX. We digested the DNA with NHeI restriction enzymes prior to amplification. The PCR reaction mix contained 0.25 ng/ul of DNA, 900 nM of each primer (target and control), and 250 nM of fluorescent probes. Droplet generation (20,000) was done by QX200 Automated Droplet Generator, and the droplets were read by QX200 Droplet Reader. Droplet counts were analyzed using QuantaSoft software, and the copy number was estimated by dividing the number of FAM-positive droplets by the number of HEX-positive droplets (Supplementary Fig. 9). For each individual, the reported copy number represents an average of three independent replicates. Given that *AMY1* and *AMY2A* share odd/even parity, we checked the correctness of the *AMY1* CN call using the *AMY2A* call, as done in ref. ^3^. For a given sample, if both *AMY1* calls obtained from two independent probes were concordant, they were averaged. If only one was concordant, only the concordant *AMY1* genotype was used. If both calls were discordant, they were averaged.

A further Independent in silico validation of *AMY1* CN in the Qatari population was performed using AMYCNE^18^ (release: 2020-03-18) which was developed particularly for genes harboring more than four copies, and its accuracy in estimating *AMY1* CN was assessed by the authors using ddPCR, making it particularly suitable for identifying *AMY1* CN variations. Copy number at *AMY1* was estimated using region files provided by AMYCNE and BAM coverage files obtained using TIDDIT^40^.

### Loss-of-function and missense variants at the *AMY1* gene

Coding-region variants at the *AMY1* gene were annotated using SNPeff^41^ which implements coding effect prediction of genetic variants. Stop gained, frameshift, missense variants or inframe deletions were considered as high-impact variants.

### *AMY1*, *AMY2A*, and adiposity traits association testing in the Qatari population

We tested the association between amylase genes CNs and adiposity traits in 2935 individuals from the QBB. Association testing was carried out using PopPAnTe^19^ (v. 1.0.2) which uses a variance component framework to model the resemblance among related individuals. Subjects showing zero CN at *AMY1* were discarded from the analysis, and rare *AMY1* CNs larger than 14 copies were set to 14. Sex, age at the sample collection, and the first 10 PCs from genome-wide data were included as covariates. The phenotypic covariance matrix between subjects was modeled using the matrix of the kinship between each pair of individuals, as evaluated by PLINK^42^. Indeed, the Qatari population is an isolated inbred population characterized by a large number of consanguineous families^43^. Pairs of individuals having a kinship lower than 0.05 were considered as unrelated (i.e., their kinship was considered to be zero).

We derived the Bonferroni-adjusted threshold for statistical significance by dividing a conventional alpha value of 0.05 by the number of tests performed, considering both the number of outcome variables (*N* = 3) and the number of predictors tested (i.e., *AMY1* and *AMY2A*; *N* = 2).

### *AMY1* and adiposity traits association testing in Arabs and Persians from Qatar

To identify the ethnicity of each of the 2935 participants from the QBB, we leveraged genotypes at 48 ancestry-informative single-nucleotide polymorphisms (SNPs) from the WGS data to differentiate the three major Qatari subpopulations (Bedouin Arabs, Persians, East Africans) as described in refs. ^20,21^. Individuals whose majority ancestry did not belong to these three major ethnicities were considered admixed.

Subsequently, we sought for *AMY1* association with adiposity traits using PopPAnTe (v. 1.0.2), in 948 Persians and 1518 Arabs from the QBB. Subjects showing zero CN at *AMY1* were discarded from the analysis, and rare *AMY1* CNs larger than 14 copies were set to 14. Sex, age at the sample collection, and the first 10 PCs from genome-wide data were included as covariates.

The Bonferroni-adjusted threshold for statistical significance was obtained by dividing a conventional alpha value of 0.05 by the number of tests performed, considering both the number of outcome variables (*N* = 3) and of groups tested (*N* = 2).

### Arabs and Persians clustering based on genotypes at the amylase region

To verify whether some degree of admixture at the amylase genomic region was not properly captured by the ethnicity-informative SNPs^20,21^ used to assign subjects to the different ethnic groups, we carried out PCA on 6,229 SNPs and indels located within a 5-MB window surrounding *AMY1* (chr1:99,159,954–109,301,314; Supplementary Fig. 4). PCA was performed using PLINK (v. 1.9) on LD-pruned high-quality variants (MAF > 0.01, genotyping rate >0.9, HWE deviation *P* > 10^−6^). LD pruning was carried on with PLINK (option: --indep-pairwise) using the following parameters: window size of 50-kb, shift of 5 variants, and r^2^ threshold of 0.05. This analysis highlighted the presence of a small number of outliers (*N*_Arabs_ = 75, *N*_Persians_ = 34, Supplementary Fig. 4). Therefore, to rule out the possibility that these individuals could possibly be skewing the association signal in our primary analysis, we removed them from our dataset, and re-assessed the association between *AMY1* CN and adiposity traits using PopPAnTe (v. 1.0.2), and including sex, age, and the first 10 PCs from genome-wide data as covariates.

### Case/control *AMY1* association with obesity in Arabs from Qatar

Association of *AMY1* CNs with obesity was assessed in Arabs from the QBB by logistic regression using R (*lme4* package, v. 1.1–21). Individuals with BMI ≥ 30 kg/m^2^ were considered as obese, whereas subjects with BMI < 25 kg/m^2^ were treated as controls. Rare *AMY1* CNs larger than 14 were set to 14. Sex, age at the sample collection and the first 10 PCs from genome-wide data were included as covariates in the analysis.

### *AMY1* association with dietary restraint

In order to explore whether ethnic-specific behavioral variations underlie inter-population variability in *AMY1* association with adiposity, we sought association between *AMY1* copy number and dietary restraint, collected through self-administered questionnaires as part of the QBB initiative.

Association of self-reported dietary restraint with *AMY1* CNs was evaluated in overweight subjects (BMI ≥ 25 kg/m^2^) of Persians or Arabs ancestry from the QBB by logistic regression in R (*lme4* package, v. 1.1–21), including age, sex, BMI, and ethnicity as covariates. Rare *AMY1* copy numbers larger than 14 were set to 14.

### Dissecting the confounding effect of ethnicity

To dissect the effect of ethnicity in our analyses, we evaluated the association between the first principal component (PC1) values and *AMY1* CNs, as well as adiposity traits. We also investigated the association between *AMY1* CNs and adiposity traits, as well as the tendency towards dietary restraint behaviors, in subsets of individuals of Arab or Persians ancestry, binned according to their PC1 values (Supplementary Fig. 10). More in detail, we generated 10 sliding windows, each including 750 Arab individuals (with an overlap of 85 individuals) along with the first principal component in Arabs. Similarly, three sliding windows, each including 750 Persian individuals (with 85 individuals of overlap) were generated along with the first principal component in Persian. Within each window, we tested the association between *AMY1* CN and BMI, total and trunk fat mass percentages using linear regression (stats R package, v. 3.6). Outliers (measurements greater than three standard deviations the dataset mean) were removed, and the data inverse normal transformed. Age and sex were included as covariates. Association with dietary restraint was assessed using logistic regression (stats R package, v. 3.6), and adjusting for sex, age, and BMI.

Heritability of the adiposity traits was estimated using PopPAnTe (v. 1.0.2), accounting for age and sex.

### Reporting summary

Further information on research design is available in the Nature Research Reporting Summary linked to this article.

## Supplementary information

Supplementary Information

Supplementary Data 1

Reporting Summary Checklist

## Data Availability

The anonymized study participants information on ancestry, estimated *AMY1* and *AMY2* copy numbers, BMI, total and trunk fat percentages (age and sex-adjusted standardized residuals) are available as Supplementary Data 1. Raw data should be requested through filling the access application at website www.qatarbiobank.org.qa and submitted to the research access office accessofficeqbb@qf.org.qa. Requests are reviewed and approved by QBB IRB and Access Committee.

## Code availability

Analyses were carried out using open-source published software which are referenced in the manuscript.
