# Supplementary material for: Ethnic-specific association of amylase gene copy number with adiposity traits in a large Middle Eastern biobank
Source: NPJ Genom Med. 2021 Feb 9;6:8. doi: 10.1038/s41525-021-00170-3 (PMC7873199; doi:10.1038/s41525-021-00170-3)
Supplement: Supplementary file 1 — Supplementary Information [file 41525_2021_170_MOESM1_ESM.pdf]

Supplementary Material for:

Ethnic-specific association of amylase gene copy number with adiposity traits in a large Middle Eastern biobank

Niccolo' Rossi[1,#], Elbay Aliyev[2,#], Alessia Visconti[1], Ammira S.A. Akil[2], Najeeb Syed[2], Waleed Aamer[2], Sujitha S. Padmajeya[2], Mario Falchi[1*], and Khalid A. Fakhro[2,3,4*]

[1] Department of Twin Research & Genetics Epidemiology, King's College London, London, UK

[2] Department of Human Genetics, Sidra Medicine, Doha, Qatar

[3] Department of Genetic Medicine, Weill-Cornell Medical College, Doha, Qatar

[4] College of Health and Life Science, Hamad Bin Khalifa University, Doha, Qatar

[#]NR and EA contributed equally

[*]KAF and MF share senior authorship

Supplementary Methods

AMY1 and *AMY2A* haplotype estimation

Haplotypes at *AMY1* and *AMY2A* loci were estimated in 948 Persians and 1,518 Arabs from the Qatari Biobank (QBB) from integer-rounded CN diplotypes inferred through coverage analysis of whole-genome sequencing data, using CNVphaserPro[1] with default parameters. CNVphaserPro uses Partition-Ligation Expectation-Maximization (PL-EM) to estimate haplotype frequencies from diploid, unphased CN genotypes. CNVphaserPro returns a posterior probability distribution over possible haplotypes given the observed genotypes. Haplotypes at *AMY1* and *AMY2A* were estimated separately in Persians and Arabs, in order to account for possible differences in haplotype frequencies between diverse ethnic groups. For each sample, we selected the haplotype with the highest probability as the inferred haplotype. Differences between the expected and observed frequencies of *AMY1* and *AMY2A* haplotypes and genotypes were assessed by χ^2 test in R (v. 3.4.4, *chisq.test* function).

Supplementary Results

We dissected the genetic architecture of *AMY1* and *AMY2A* in Arabs and Persians by estimating the frequencies of haplotypes at the two genes in the two ethnic groups using the Partition-Ligation Expectation-Maximization (PL-EM) algorithm, as implemented in CNVphaserPro[1], on inferred *AMY1* and *AMY2A* CNs. All Arabs and Persians carrying no CNs of *AMY2A* were estimated to have two *AMY1*-odd haplotypes, whereas all carriers of one *AMY1*-even haplotype displayed a non-diploid copy number of *AMY2*. This analysis suggested that the observed differences in *AMY1* and *AMY2A* CNs distribution between Arabs and Persian were supported by differences in haplotypes frequencies at both loci (χ^2 test $P<2.62x10^{-22}$), with Persians carrying overall higher allelic copy numbers at both *AMY1* ($\mu_{Arabs}=3.5$; $\mu_{Persians}=3.8$; Wilcoxon's test $P=1.61x10^{-13}$; **Supplementary Fig. 4)** and *AMY2A* ($\mu_{Arabs}=1.03$; $\mu_{Persians}=1.14$; Wilcoxon's test $P=1.76x10^{-22}$; **Supplementary Fig. 4**).

Supplementary References

1. Kato, M. *et al.* Inferring Haplotypes of Copy Number Variations From High-Throughput Data With Uncertainty. *G3amp58 GenesGenomesGenetics* **1**, 35–42 (2011).

Supplementary Data

Supplementary Data 1. For each anonymized study participant, we provide information on ancestry, estimated *AMY1* and *AMY2* copy numbers, BMI, total and trunk fat percentages (age and sex-adjusted standardised residuals).

Supplementary Tables

Supplementary Table 1. Independent validation of estimated copy numbers of *AMY1* and *AMY2A* by ddPCR on 40 QBB samples. For each sample, we report raw (Probe) and integer (consensus) ddPCR CN estimates as obtained using one (*AMY2A*) or two independent probes (*AMY1*), as described in Usher *et al.* [doi: 10.1038/ng.3340], and CN estimated from sequencing data using the method described by Carpenter *et al.* [doi: 10.1093/hmg/ddv098]. Samples where Carpenter's calls deviate by a single copy from ddPCR results are highlighted in yellow, whereas deviations >1 copy are highlighted in red.

	AMY1				***AMY2A***		
	**ddPCR**				**ddPCR**		
**Sample**	**Probe (1)**	**Probe (2)**	**consensus**	**Carpenter**	**Probe**	**consensus**	**Carpenter**
1	6.07	6.03	6	6	1.82	2	2
2	5.70	5.71	6	6	2.02	2	2
3	4.07	4.01	4	4	1.95	2	2
4	7.86	7.99	8	8	2.04	2	2
5	8.17	7.72	8	8	2.00	2	2
6	3.91	3.85	4	4	1.99	2	2
7	3.05	3.30	3	3	1.03	1	1
8	12.00	11.00	12	12	1.98	2	2
9	7.22	6.26	7	7	3.03	3	3
10	14.40	12.90	14	14	2.00	2	2
11	8.18	7.98	8	5	1.92	2	1
12	3.80	3.62	4	4	1.98	2	2
13	9.70	8.07	9	10	2.05	2	2
14	5.75	5.65	6	6	1.96	2	2
15	9.91	9.80	10	10	2.06	2	2
16	12.10	12.00	12	12	2.00	2	2
17	12.50	11.20	12	12	2.00	2	2
18	11.10	10.90	11	11	1.00	1	1
19	11.60	11.10	12	12	1.96	2	2
20	11.90	10.90	12	12	1.98	2	2
21	7.80	8.00	8	8	2.02	2	2
22	6.91	6.70	7	7	0.97	1	1
23	8.08	7.80	8	8	2.00	2	2
24	9.88	9.50	10	10	1.98	2	2
25	5.80	6.00	6	6	3.97	4	4
26	14.10	13.80	14	14	2.14	2	2
27	11.80	9.30	12	12	1.90	2	2
28	9.27	9.30	9	9	2.92	3	3
29	7.70	6.70	8	8	2.00	2	2
30	5.93	5.40	6	6	2.07	2	2
31	4.96	4.60	5	5	0.95	1	1
32	3.90	3.64	4	4	1.89	2	2
33	4.90	5.14	5	5	3.04	3	3
34	14.10	11.90	13	14	1.97	2	2
35	11.80	11.20	12	12	4.00	4	4
36	9.80	8.40	9	10	1.98	2	2
37	8.06	7.23	8	8	2.04	2	2
38	5.18	4.60	5	5	2.90	3	3
39	3.90	3.70	4	4	1.90	2	2
40	12.50	10.10	11	12	2.09	2	2

Supplementary Table 2. Association between *AMY1* CN and adiposity measurements in Arabs and Persians from Qatar, clustered according to 6,229 SNPs and indels located within a 5-Mb window surrounding the *AMY1* gene. Association testing was performed using PopPAnTe, which takes into account the kinship between individuals, and including age, sex, and first 10 PCs from genome-wide data as fixed effects. The table reports the number of samples included in the tested datasets (N), the effect sizes (β) with their standard error (SE), and the association p-values (P). Association results from our primary analysis are also reported, to facilitate the comparison. P-values below the Bonferroni-corrected significance threshold ($P<8.33x10^{-3}$) are highlighted in bold.

		**Primary results**				***Results based on regional clustering around AMY1***			
**Ancestry**	**Trait**	**N**	**β**	**SE**	**P**	**N**	**β**	**SE**	**P**
Arabs	BMI	1,505	-0.03	0.01	**$4.60x10^{-3}$**	1431	-0.03	0.01	**$6.90x10^{-3}$**
	Total fat (%)	1,432	-0.02	0.01	**$8.40x10^{-3}$**	1361	-0.02	0.01	$9.70x10^{-3}$
	Trunk fat (%)	1,425	-0.03	0.01	**$4.40x10^{-3}$**	1356	-0.02	0.01	**$6.90x10^{-3}$**
Persians	BMI	944	0.00	0.01	0.80	910	-0.01	0.02	0.54
	Total fat (%)	879	-0.01	0.01	0.30	847	-0.01	0.01	0.30
	Trunk fat (%)	874	-0.01	0.01	0.32	842	-0.01	0.01	0.33

Supplementary Table 3. ddPCR primers and probes.

	Primer[1]	Primer[1]	Probe[1]	Reverse probe[2]	Tag	$T_{annealing}$
**AMY2A_assay1**	AACATCAAAAAGTCTCTC ATGGAA	CAAATTTTGGTTTTCTACT GTTATGTG	GGCCCCAGCAACAGGTCA CTG	CAGTGACCTGTTGCTGGG GCC	FAM	57° C
**AMY1_assay1**	AAAAACCCAAGAATTAGG AATGG	CCTGGAAGGATTTTCTGGT G	TGCTCTCATTTTTAGATGA CTTGTG		FAM	55° C
**AMY1_assay2**	TGTTTGCAAGGAGGTCTTC TC	TTGGCCTTTCATCTGTGAT TT	AAATGATTCCCGAAACTG TAGC		FAM	55° C
**Controls**	AAATTTATTGGAGGGATG TTGG	TTCAAGTTTGACTGCTAAC TCCTG	TGGAATAAAGAATCATTG GGCACAGGT		HEX	

[1]Obtained from Usher *et al.* [doi: 10.1038/ng.3340]

[2]Additional reverse complementary sequence was used to avoid quenching of FAM signal by the 1st "G" nucleotide.

Supplementary Figures

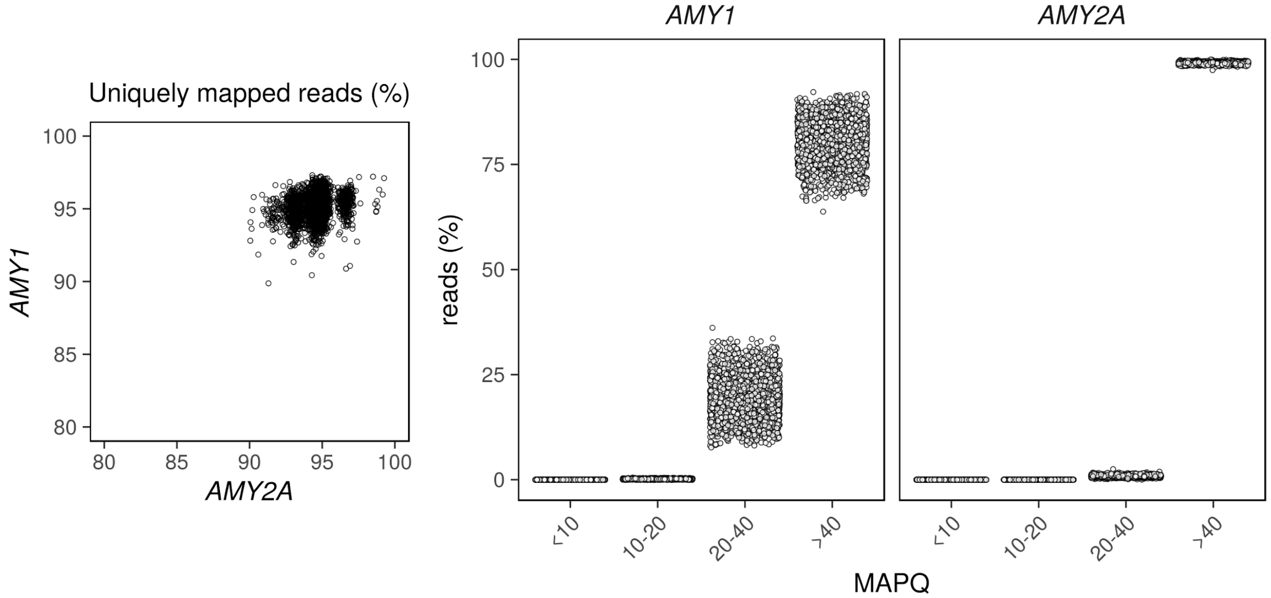

Supplementary Figure 1. Misalignment rate of reads mapping to the *AMY1* and *AMY2A* genes. **Left panel**: Ratio (expressed as a percentage) between properly paired reads with a unique mapping *vs* total number of aligned reads in proper pairs, with respect to the *AMY1* or *AMY2A* gene. **Right panel:** Distribution of reads mapping to either *AMY1* or *AMY2A* according to mapping quality (MAPQ score), which is defined as the log-scaled probability that the mapping is inaccurate and ranges from 0 (Pr=1) to 60 (Pr=$1x10^{-6}$).

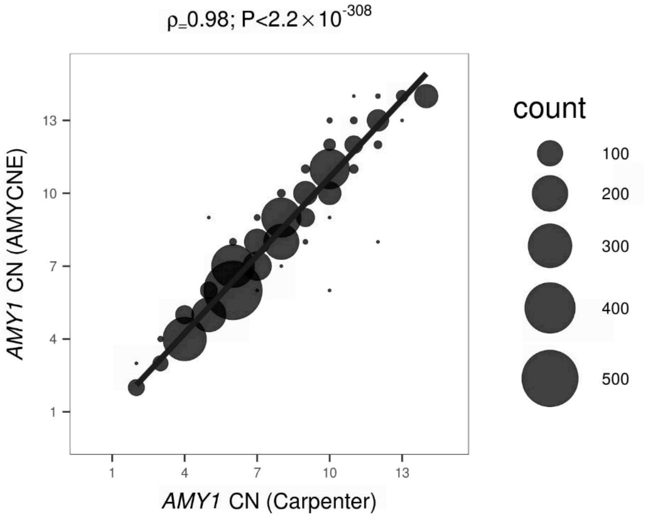

Supplementary Figure 2. **Concordance of *AMY1* CN estimates.** The method described by Carpenter *et al.* [doi: 10.1093/hmg/ddv098] and AMYCNE [doi: 10.1371/journal.pone.0189710] were used to estimate *AMY1 CN* in 2,935 individuals from Qatar.

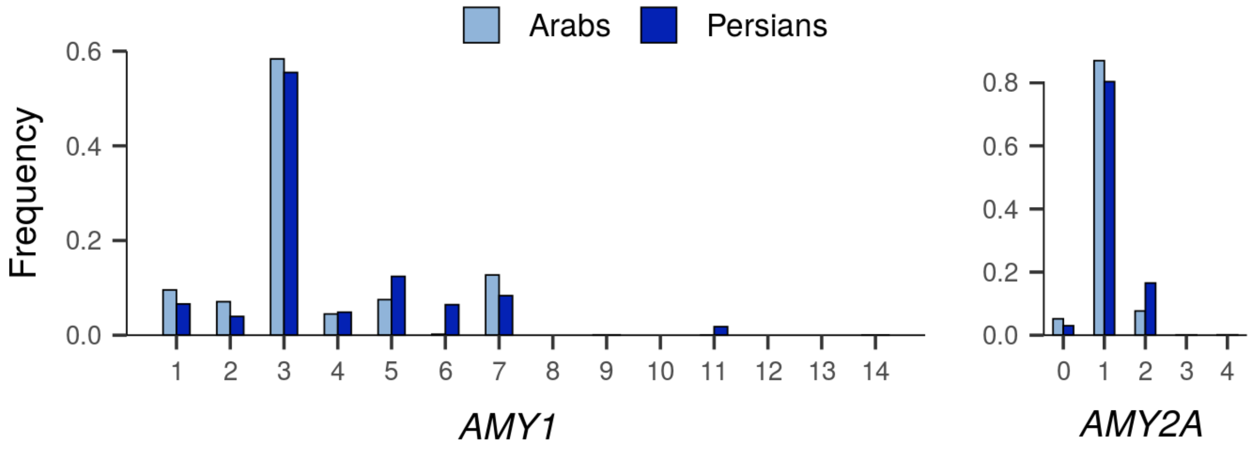

Supplementary Figure 3. *AMY1* and *AMY2A* haplotypes variation in the Qatari population. The histograms show the distribution of estimated allelic copy numbers of *AMY1* and *AMY2A* in Arabs (light blue) and Persians (dark blue) from Qatar, obtained from coverage analysis of whole-genome sequencing data. Persians tend to have higher allelic copy numbers at both *AMY1* and *AMY2A* ($P<1.61x10^{-13}$).

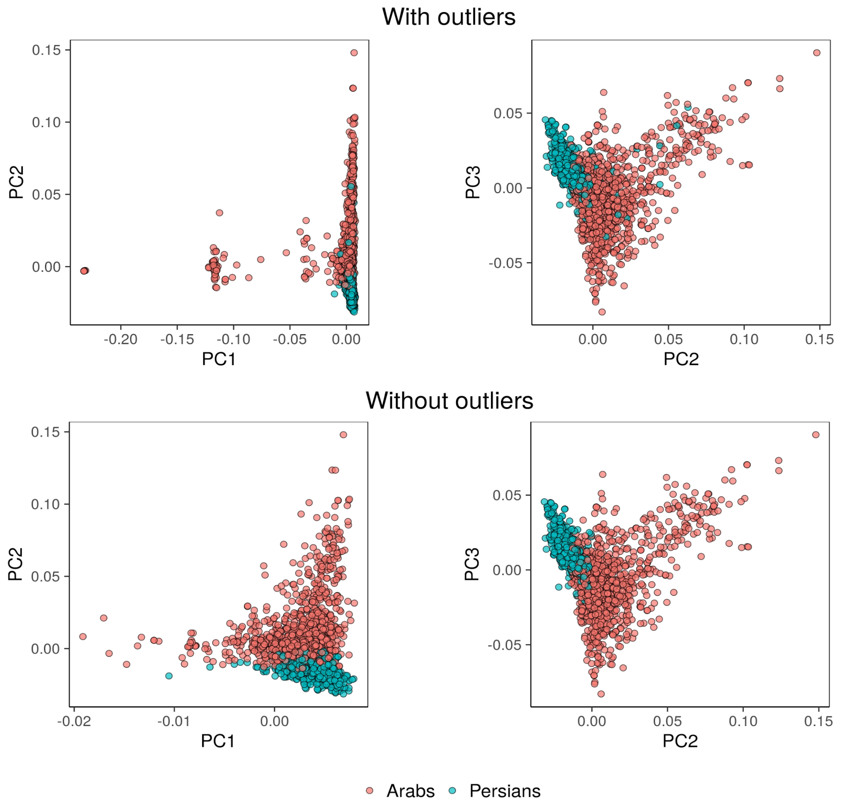

Supplementary Figure 4. Arabs and Persians clustering based on the *AMY1* region. Scatterplot of the first three principal components assessed on 6,229 SNPs and indels of Arabs and Persians from Qatar located within a 5-MB window surrounding the *AMY1* gene. Individual data points are colored according to ethnic ancestry defined in our primary analysis according to 48 ancestry-informative SNPs [doi: 10.1186/s12864-015-1991-5]: Arabs are in red, Persians in cyan. Individuals with PC1< -0.025 were defined as outliers.

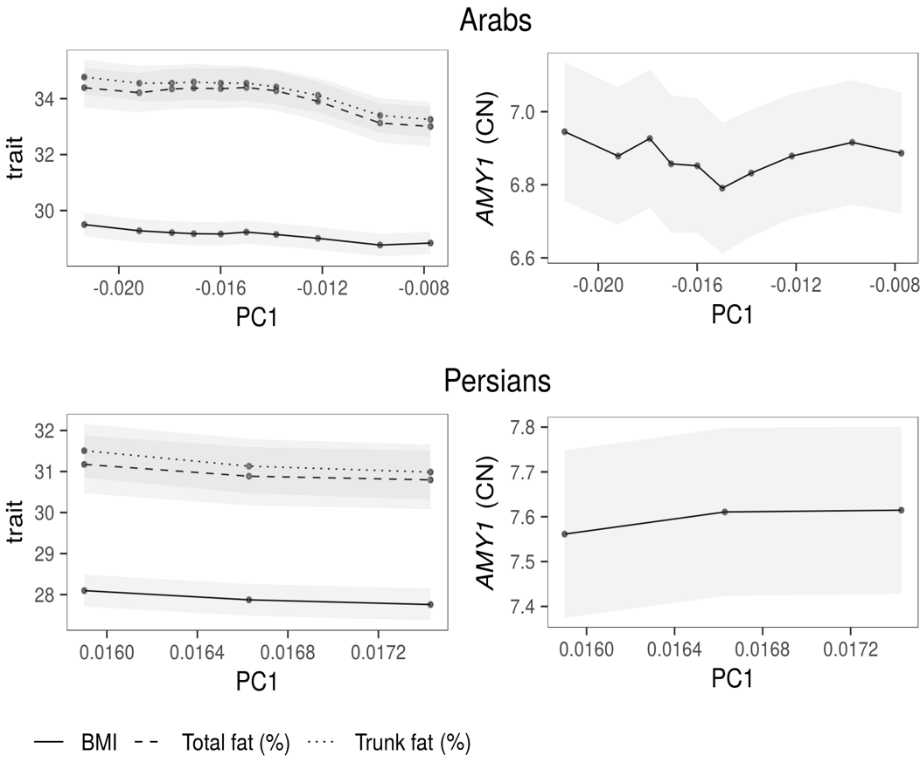

Supplementary Figure 5. Distribution of *AMY1* CN and adiposity measures as a function of the degree of ancestry in Arabs and Persians from Qatar. Within each plot, a dot shows median PC1 value, estimated within subsets of 750 individuals (with an overlap of 85 individuals) binned according to PC1 value, and corresponding mean BMI, total and trunk fat percentages value (**left plots**), or *AMY1* CN (**right plots**), as evaluated in the same subset of individuals. Sex and age were included as covariates. Grey areas indicate 95% confidence intervals.

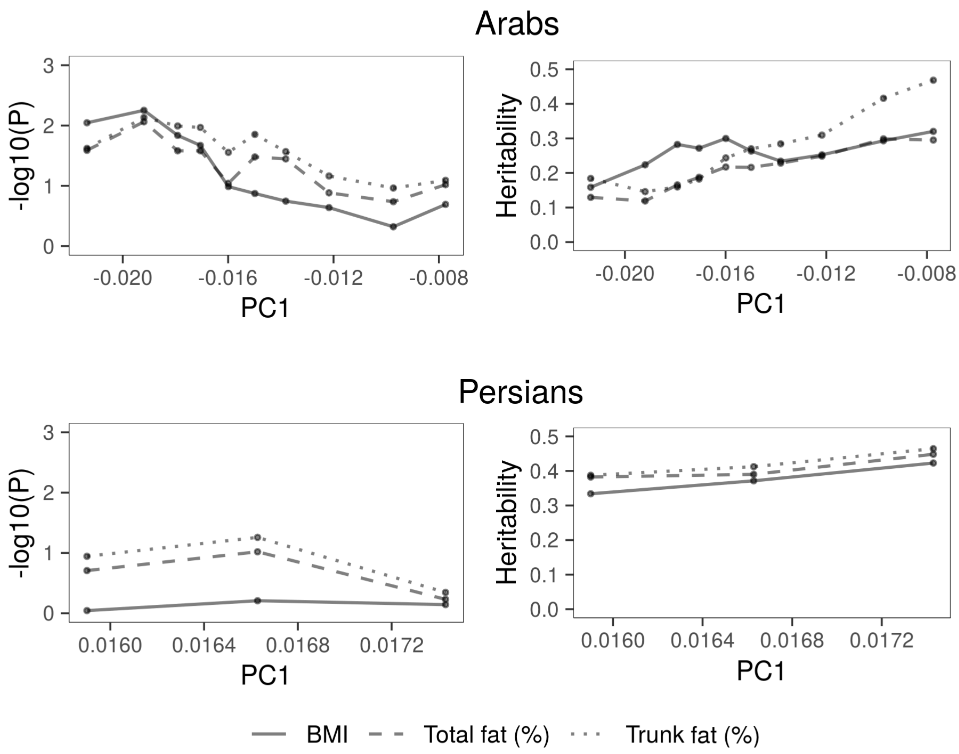

Supplementary Figure 6. Association between *AMY1* CN and adiposity measures as a function of the degree of ancestry in Arabs and Persians from Qatar. Within each plot, a dot shows median PC1 value, estimated within subsets of 750 individuals (with an overlap of 85 individuals) binned according to PC1 value, and corresponding p-value of the association between *AMY1* CN and adiposity measures (**left plots**), or adiposity traits heritability (**right plots**), as evaluated within the same subset of individuals. Sex and age were included as covariates.

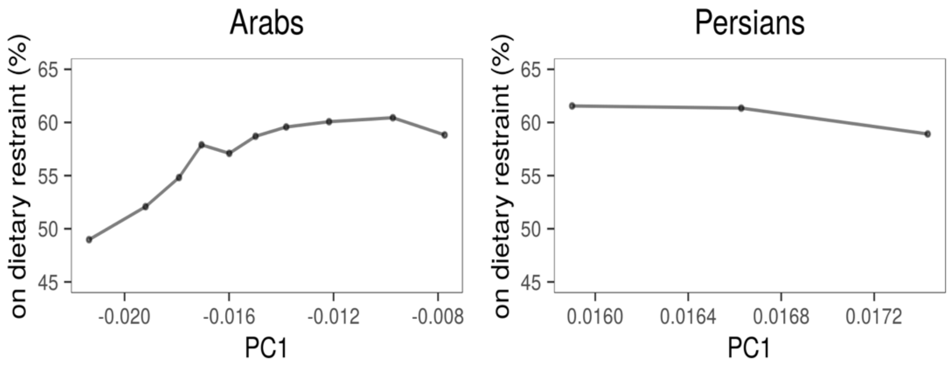

Supplementary Figure 7. Dietary restraint prevalence as a function of the degree of ancestry. Within each plot, a dot shows median PC1 value, estimated within subsets of 750 individuals (with an overlap of 85 individuals) binned according to PC1 value, and corresponding prevalence, expressed as a percentage, of dietary restraint behavior among overweight subjects (BMI ≥ 25 kg/m^2) from the same subset.

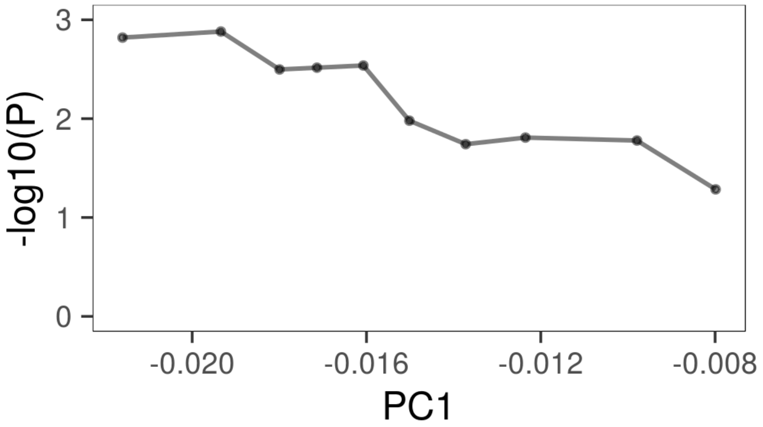

Supplementary Figure 8. Association between *AMY1* CN and dietary restraint behavior as a function of the degree of ancestry. A dot shows median PC1, estimated within subsets of 750 individuals (with an overlap of 85 individuals) binned according to PC1 value, and corresponding p-value of the association between *AMY1* CN and dietary restraint among overweight subjects (BMI ≥ 25 kg/m^2) from the same subset. Sex, age and BMI were included as covariates.

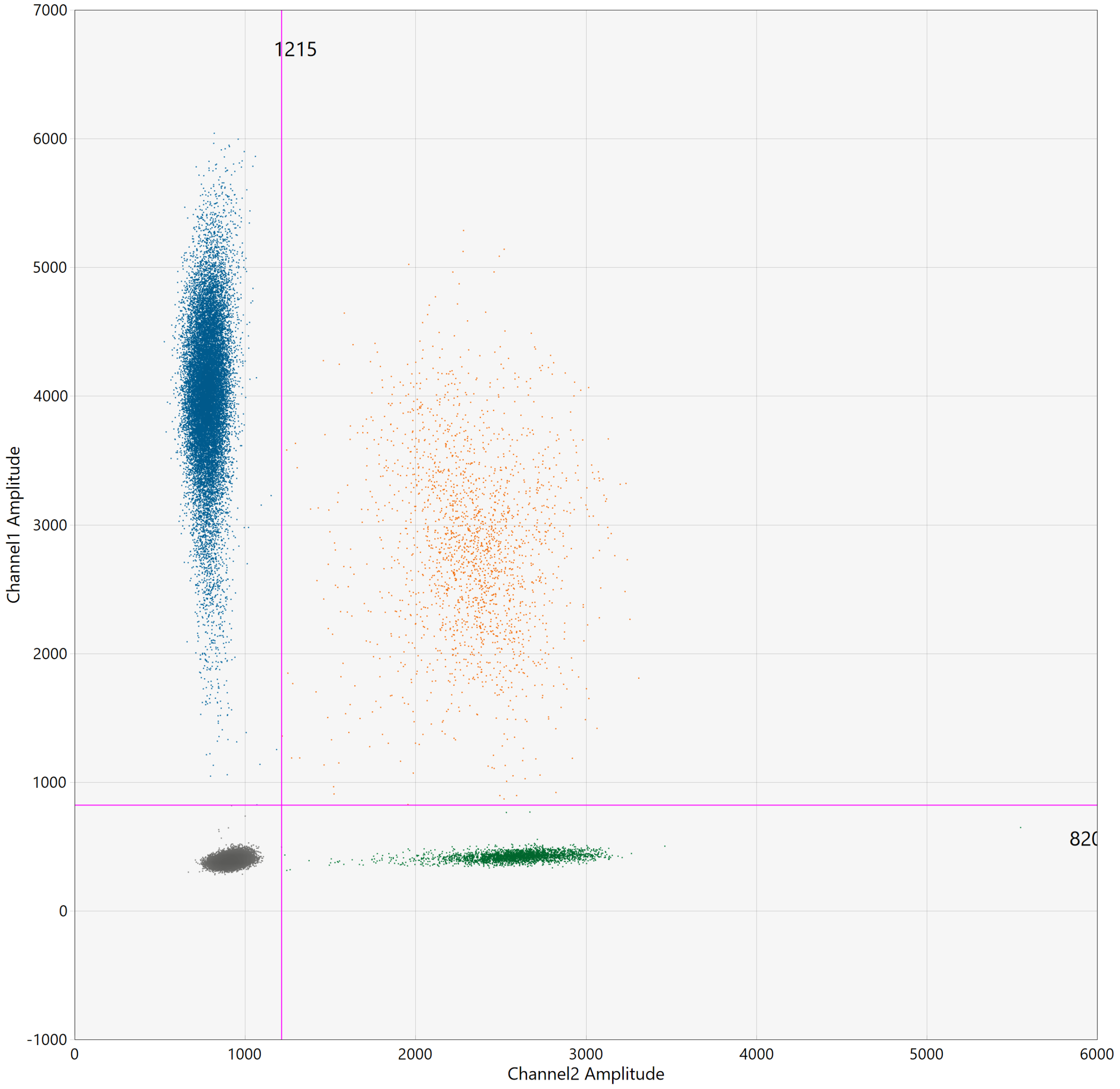

Supplementary Figure 9. ddPCR. Screenshot from the QuantaSoft program showing a QBB sample analyzed for AMY1 CN *(AMY1_assay1)*. Thresholds for calling are drawn in pink. On the x-axis is HEX fluorescence, and on the y-axis is FAM. Each dot represents a droplet.

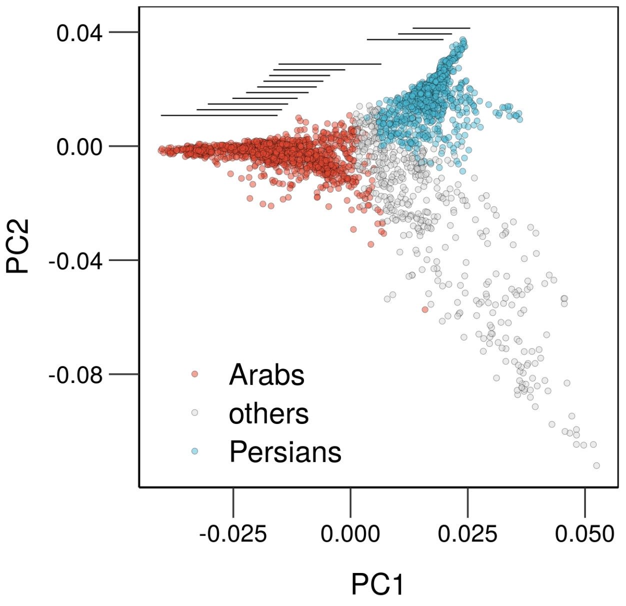

Supplementary Figure 10. Arabs and Persians binning according to PC1. Scatterplot of the first two principal components highlighting individuals of Arab (red) and Persian (cyan) ancestry. Horizontal lines show the PC1 range used to bin the cohort into sliding windows of 750 individuals (with an overlap of 85 individuals).
