# Supplementary material for: Ethnic-specific association of amylase gene copy number with adiposity traits in a large Middle Eastern biobank
Source: NPJ Genom Med. 2021 Feb 9;6:8. doi: 10.1038/s41525-021-00170-3 (PMC7873199; doi:10.1038/s41525-021-00170-3)
Supplement: Supplementary file 3 — Reporting Summary Checklist [file 41525_2021_170_MOESM3_ESM.pdf]

nature research

Corresponding author(s): Dr Khalid Fakhro

Last updated by author(s): Dec 17, 2020

Reporting Summary

Nature Research wishes to improve the reproducibility of the work that we publish. This form provides structure for consistency and transparency in reporting. For further information on Nature Research policies, see our Editorial Policies and the Editorial Policy Checklist.

Statistics

For all statistical analyses, confirm that the following items are present in the figure legend, table legend, main text, or Methods section.

n/a	Confirmed	
☐	☒	The exact sample size (*n*) for each experimental group/condition, given as a discrete number and unit of measurement
☐	☒	A statement on whether measurements were taken from distinct samples or whether the same sample was measured repeatedly
☐	☒	The statistical test(s) used AND whether they are one- or two-sided<br>*Only common tests should be described solely by name; describe more complex techniques in the Methods section.*
☐	☒	A description of all covariates tested
☐	☒	A description of any assumptions or corrections, such as tests of normality and adjustment for multiple comparisons
☐	☒	A full description of the statistical parameters including central tendency (e.g. means) or other basic estimates (e.g. regression coefficient) AND variation (e.g. standard deviation) or associated estimates of uncertainty (e.g. confidence intervals)
☐	☒	For null hypothesis testing, the test statistic (e.g. *F*, *t*, *r*) with confidence intervals, effect sizes, degrees of freedom and *P* value noted<br>*Give P values as exact values whenever suitable.*
☒	☐	For Bayesian analysis, information on the choice of priors and Markov chain Monte Carlo settings
☒	☐	For hierarchical and complex designs, identification of the appropriate level for tests and full reporting of outcomes
☐	☒	Estimates of effect sizes (e.g. Cohen's *d*, Pearson's *r*), indicating how they were calculated

Our web collection on statistics for biologists contains articles on many of the points above.

Software and code

Policy information about availability of computer code

Data collection	No software was used
Data analysis	- AMYCNE [DOI:10.1371/journal.pone.0189710] was used to verify the AMY copy number calls (release 2020-03-18 , source code: https://github.com/J35P312/AMYCNE)<br>- QuantaSoft Analysis Pro was used to analyse the ddPCR data (version 1.0.596)<br>- PopPAnTe [DOI: 10.1186/s12864-017-3527-7] was used for the association study and to estimate heritability of the adiposity measurements (version 1.0.2, source code: https://github.com/alesssia/poppante)<br>- R statistical environment was used for all other analysis (version: 3.6, website: https://cran.r-project.org/)

For manuscripts utilizing custom algorithms or software that are central to the research but not yet described in published literature, software must be made available to editors and reviewers. We strongly encourage code deposition in a community repository (e.g. GitHub). See the Nature Research guidelines for submitting code & software for further information.

Data

Policy information about availability of data

All manuscripts must include a data availability statement. This statement should provide the following information, where applicable:

- Accession codes, unique identifiers, or web links for publicly available datasets
- A list of figures that have associated raw data
- A description of any restrictions on data availability

The anonymized study participants' information on ancestry, estimated AMY1 and AMY2 copy numbers, BMI, total and trunk fat percentages (age and sex-adjusted standardised residuals) are available as Supplementary Data 1. Raw data should be requested through filling the access application at website

www.qatarbiobank.org.qa and submitted to the research access office accessofficeqbb@qf.org.qa. Requests are reviewed and approved by QBB IRB and Access Committee.

Field-specific reporting

Please select the one below that is the best fit for your research. If you are not sure, read the appropriate sections before making your selection.

☒ Life sciences ☐ Behavioural & social sciences ☐ Ecological, evolutionary & environmental sciences

For a reference copy of the document with all sections, see nature.com/documents/nr-reporting-summary-flat.pdf

Life sciences study design

All studies must disclose on these points even when the disclosure is negative.

Sample size	Sample study was not statistically predetermined.
Data exclusions	Subjects assigned to East African ancestry were not included in the analysis, due to their small number (n=63). Admixed individuals were excluded from the analysis, due to unassigned ancestry (n=406).
Replication	Not relevant for this study.
Randomization	Not relevant for this study.
Blinding	Not relevant for this study.

Reporting for specific materials, systems and methods

We require information from authors about some types of materials, experimental systems and methods used in many studies. Here, indicate whether each material, system or method listed is relevant to your study. If you are not sure if a list item applies to your research, read the appropriate section before selecting a response.

Materials & experimental systems

n/a	Involved in the study	
☒	☐	Antibodies
☒	☐	Eukaryotic cell lines
☒	☐	Palaeontology and archaeology
☒	☐	Animals and other organisms
☐	☒	Human research participants
☒	☐	Clinical data
☒	☐	Dual use research of concern

Methods

n/a	Involved in the study	
☒	☐	ChIP-seq
☒	☐	Flow cytometry
☒	☐	MRI-based neuroimaging

Human research participants

Policy information about studies involving human research participants

Population characteristics	Subjects included 2,935 volunteers belonging to the Qatar Biobank (1,471 females and 1,464 males, mean age 39 years old).
Recruitment	The Qatar Biobank (QBB) is a national population-based medical health initiative started in 2012 with the aim of collecting samples and information on health and lifestyle from 60,000 Qatari adult nationals and long-term residents [doi: 10.1186/s12889-015-2522-7]
Ethics oversight	The Qatar Biobank study was approved by the Qatar Biobank Institutional Review Board (IRB) Committee (IRB protocol E/2017/RES-ACC-0032/0002).

Note that full information on the approval of the study protocol must also be provided in the manuscript.
